# Anorexia and Eating Patterns in the Elderly

**DOI:** 10.1371/journal.pone.0063539

**Published:** 2013-05-02

**Authors:** Lorenzo Maria Donini, Eleonora Poggiogalle, Maria Piredda, Alessandro Pinto, Mario Barbagallo, Domenico Cucinotta, Giuseppe Sergi

**Affiliations:** 1 Department of Experimental Medicine, Medical Physiopathology, Food Science and Endocrinology Section, “Sapienza” University of Rome, Italy; 2 Department of Clinical Medicine and Emerging Pathologies, Geriatric Unit, University of Palermo, Italy; 3 Department of Clinical Medicine and Aging, S. Orsola-Malpighi Hospital, Bologna, Italy; 4 Department of Medical and Surgical Sciences, Geriatrics Section, University of Padua, Italy; St. Joseph's Hospital and Medical Center, United States of America

## Abstract

**Objectives:**

To evaluate the change in eating habits occurring in community- dwelling and institutionalized elderly subjects with senile anorexia.

**Design:**

Cross- sectional, observational.

**Setting:**

Community, nursing homes and rehabilitation or acute care facilities in four Italian regions.

**Participants:**

**A** random sample of 526 subjects, aged 65 years and older (217 free living individuals, 213 residents in nursing homes, and 93 patients in rehabilitation and acute wards).

**Measurements:**

All subjects underwent a multidimensional geriatric evaluation of: nutritional status, anthropometric parameters, health and cognitive status, depression, taste, chewing and swallowing function, and some hormones related to appetite. Diet variety was assessed, considering the frequency of consumption of different food groups (milk and dairy products; meat, fish, and eggs; cereals and derivatives; fruit and vegetables).

**Results:**

In anorexic elderly subjects the global food intake was reduced, and the eating pattern was characterized by the reduced consumption of certain food groups (“meat, eggs and fish” and “fruit and vegetables”) whereas the frequency of consumption of milk and cereals remained almost unchanged. Nutritional parameters were significantly better in normal eating subjects and correlated with diet variety.

**Conclusion:**

Because of the high prevalence of senile anorexia in the geriatric population and its impact on the nutritional status, further research should be prompted to establish an intervention. protocol allowing the early diagnosis of anorexia of aging, aimed at identifying its causes and at optimizing treatment of anorexic patients.

## Introduction

In the elderly the main change in the eating behavior affects the “food choice”, that is influenced by the interplay of several factors: biologic aspects (satiety signals depending on single macronutrients and food energy density), palatability (including food texture, taste, olfaction, and sight stimuli), economic factors, social features (access to food, autonomy level, education degree, social environment, familial support) as well as psychological factors (depression, stress) [1].

In older persons the most frequent change in the eating behavior is the anorexia of aging, that is usually classified as: physiologic, pathologic, environmental, and psychological anorexia of aging [2].

Physiologic anorexia may be due, in particular, to age- related alterations affecting the gastro-intestinal system (impaired chewing ability, reduced function of salivary glands, impaired esophageal motility, decreased gastric secretion, reduced intestinal absorptive surface), decreased adaptive relaxation of the fundus of the stomach and increased effectiveness of cholecystokinin (CCK) action, impairment of the central feeding drive (underlying the opioid system and the neuropeptide Y signaling) and decrement of taste and smell (due to loss of sensitivity, the decrease in the number of gustative papillae, poor oral hygiene) [3]–[11].

“Pathologic” anorexia is a consequence of intercurrent illnesses like cancer (as cytokines deriving from tumor tissue may act as important anorexing agents), chronic pulmonary obstructive disease (difficult respiration during the meal consumption), cerebral ischemic attacks (difficulty swallowing), abdominal angina (severe abdominal pain after a meal), chronic constipation (causing a feeling of fullness), dementia (indifference to food) and depressive syndromes (anorexia might be linked to the increase of the hypothalamic corticotropin releasing factor). This subtype of anorexia is mostly diagnosed in institutionalized elderly [12]–[15]. Moreover, anorexia in the elderly may be an iatrogenic effect of pharmacologic treatments, or it may occur after admission to hospital [14]. Also social issues (poverty, solitude, social isolation) and psychological factors (like depression, often associated to role loss and decline of social status) [15]–[17].

Finally, a food intake lower than the recommended daily requirements is common among elderly people. This eating behavior is present in almost 30% of the community dwelling elderly persons whose age ranges from 70 and 80 years, living independently in their own home. This phenomenon is more severe when considering older subjects living in nursing homes or in residential care settings. The “Euronut- Seneca” Survey reported that one third of elders living autonomously in their homes showed a deficient intake of at least one micronutrient, and in more than 4% protein- calorie malnutrition was described; the latter increased till 85% as taking into account institutionalized elderly people. Furthermore, malnourished older adults are at risk of increased morbidity and mortality [18]–[19].

In previous papers we reported the results of a study that we carried out in order to estimate the prevalence and causes of anorexia in a population of Italian elderly people, living in four regions (Lazio, Sicily, Emilia- Romagna and Veneto) in three different settings (free- living older people, seniors living in nursing homes, and in rehabilitation and acute wards) [20], [21].

The present study was aimed to assess changes in the eating pattern in elderly subjects with anorexia.

## Methods

### Ethics statement

The study was performed after the approval of the “Azienda Policinico Umberto I” of Rome ethics committee. Oral and written informed consent was obtained from participants or their legally authorized representatives.

From April 2006 to June 2007, a sample of 526 over 65- year- old subjects was recruited (218 free- living persons, 213 subjects from long-term care nursing homes, and 96 subjects from rehabilitation wards or acute geriatric wards) from four Italian facilities located in Rome (Clinical Rehabilitation Institute “Villa delle Querce”, Nemi, Lazio), Bologna (S. Orsola- Malpighi Hospital, Emilia- Romagna), Padua (Department of Medical and Surgical Sciences, Geriatrics Section, Veneto), and Palermo (Geriatrics Unit, Department of Internal Medicine and Emergent Pathologies, Sicily). This study was performed as a part of a Research Project of National Interest (PRIN) sponsored by the Italian Ministry of Education, University and Research (MIUR).

All the subjects present in the selected settings during the study period (April 2006 – June 2007), and respecting inclusion and exclusion criteria, were recruited.

At the time of recruitment, food intake was recorded over three days using the “*Club Francophone de Gériatrie et Nutrition*” form [22]; at the end of each meal dietitians (in long- term care nursing homes, rehabilitation wards and acute geriatric wards) and caregivers, for free- living subjects, directly estimated the portion of food eaten and quantified it in quarters (0 to 4/4) compared to a standard whole meal. The diagnosis of anorexia was considered when a reduction in food intake, equal or greater than 50% of the daily intake of food, was recorded for at least three days, in case of absence of disorders preventing mastication (i.e. dysphagia, oral pain). The daily amount of energy and nutrients, and the dimension of servings for each food were based on Italian Recommended Daily Allowances (LARN: Levels of Recommended Intake of Energy and Nutrients) [23].

Exclusion criteria included: 1) patients requiring parenteral and/or enteral nutrition; 2) patients with medical conditions precluding a reliable nutritional assessment: severe liver failure (Child- Pugh class C), renal failure (GFR <30 mL/min/1.73m^2^), and/or heart failure (NYHA class IV), presence of severe oedema) or affected by severe comorbidity (grade 4: severely incapacitating or life- threatening conditions) according to the Cumulative Illness Rating Scale (CIRS) [24].

All subjects underwent a multidimensional evaluation.

1. Nutritional status was assessed taking into account the following parameters:

- Mini Nutritional Assessment score [25];
- Anthropometric parameters: body mass index (BMI: weight in kilograms divided by height in meters squared), triceps skinfold thickness (TSF), arm circumference (AC), arm muscle circumference (AMC). The anthropometric measurements were performed according to the Standard Manual for Anthropometric Measures [26];
- Laboratory parameters: prealbumin, albumin, transferrin, C- reactive protein (CRP), and total cholesterol levels were measured;
- Diet variety: participants' food daily intake and frequency of food group consumption were compared to the recommended daily intake as indicated in the National Italian guidelines by the Italian National Institute of Nutrition (INRAN) [27]. Diet variety was measured considering food groups (milk and dairy products; meat, fish, and eggs; cereals and derivatives; fruit and vegetables) that were present daily in the participants' diet. A qualitatively adequate variety was established when:
- one serving of “milk and dairy products” and one serving of “meat, fish, and eggs” food groups were consumed at least once a day;
- one serving of “cereals and derivatives” and one serving of “fruit and vegetables” food groups were consumed at least twice a day.

As described before the dimension of servings for each food were based on the Italian Recommended Daily Allowances (LARN: Levels of Recommended Intake of Energy and Nutrients) [23].

2. Health status: clinical status, comorbidity and severity levels were assessed using the CIRS [24]. This scale classifies comorbidity evaluating 13 organ systems and grades each condition from 0 (corresponding to healthy organ or system) to 4 (severely incapacitating or life- threatening condition). The comorbidity index is given by the number of clinical conditions graded as ≥3. The severity index is the mean value of the severity scores of all the 13 organ systems.

Gastrointestinal symptoms potentially affecting food intake were registered as follows: constipation (weekly frequency of bowel movements), diarrhea (yes/no) and complaints of epigastric pain (yes/no).

Medications taken by the patients were examined for their impact on anorexia and malnutrition.

3. Depression was evaluated using a subjective scale, the Geriatric Depression Scale [28] and an objective scale, the Cornell Scale for Depression [29].

4. Functional and cognitive impairment were assessed using the Activity Daily Living (ADL) test [30], the Instrumental Activities of Daily Living (IADL) test [31] and Mini Mental State Examination (MMSE) [32].

5. Taste, swallowing and chewing functions:

- a research staff member counted the number of natural teeth;
- taste: subjects were asked if they had a different perception of the taste of foods from the usual taste [20];
- swallowing function was determined through a water-swallowing test [33]. This test was performed with the participant sitting on a chair. Participants were instructed to open the mouth, and 5 mL of water was instilled on the tongue using the disposable 20 mL syringe. Participants were asked to close their mouth and to swallow the contents naturally following the signal. Saturation of peripheral oxygen (SpO2) was measured using a pulse oximeter before and two minutes after swallowing;
- masticatory function: subjects were asked to chew a color- changeable chewing gum; a color scale was developed, corresponding to a numerical score ranging from 1 to 8 to evaluate the degree of color mixing, similarly with a method proposed by Hayakawa et al. [34].

6. Hormones and cytokines implicated in hunger control: ghrelin, leptin and interleukin- 6 (IL- 6) levels were measured. The quantitative measurement of serum leptin levels was performed using a leptin ELISA kit (Diagnostics Biochem Canada); ghrelin plasma concentrations were assessed with a ghrelin ELISA kit (BioVendor, Czech Republic). All measurements were performed at the laboratory at “Sapienza” University of Rome.

### Statistic analysis

After evaluating the normal distribution of data, statistical analysis was performed using the independent t-test and the analysis of variance (ANOVA) to describe differences between means of the groups, and chi-square test was used to compare observed and expected frequencies.

Variables univariately correlated with the outcome variable (presence af anorexia and diet variety defined through the number of food groups regularly consumed) were entered a pool of potential contributors in multiple regression analysis. We estimated models using a forward likelihood stepwise method (cut- off probability for entry: 0.05). With each added variable the discriminant function was recalculated and any variable that no longer met the significance level was removed from the equation (cut- off probability for removal: 0.1). Some variables with similar biological significance were excluded from the logistic analysis, in order to avoid the confounding effect of collinearity (verified with Pearson's r, t- test or χ^2^). The best fitting model was chosen according to the value of the correlation coefficients R^2^, (comparing the explained variance of the model's predictions with the total variance of the data), the adjusted R^2^ (R^2^ adj), considering a correction for inclusion of variables.

Differences were considered to be statistically significant at p<0.05. Statistical analysis was performed using SPSS 10.0 statistical software (SPSS Inc Wacker Drive, Chicago, IL, USA).

## Results

From April 2006 to June 2007, 526 subjects – 319 women (mean age: 78.4±8 years) and 208 men (mean age: 77.3±8 years) – were recruited from three settings: free- living subjects, individuals living in nursing homes, and patients from rehabilitation or acute wards. Globally, prevalence of anorexia was 21.2% (table 1), and it was higher in women than men, overall in hospital or institutional settings (33.3 versus 26.7% in rehabilitation wards, and 34.1 versus 27.2% in nursing homes, respectively).

**Table 1:** Characteristics of the Study Participants§.

		Rehabilitation and acute wards		Nursing homes		Free living subjects		
		M	F	M	F	M	F	
**No. of Subjects**		30	66	81	132	97	121	
**Age**		81.8±8	81.5±7	77.7±9	78.8±10	75.6±6	76.2±7	*
**Anorexia prevalence (%)**		26.7	33.3	27.2	34.1	11.3	3.3	*
**Clinical status**	**Comorbidity index**	3.4±2	2.6±2	2.8±2	2.3±1	1.5±1	1.7±1	*
	**Severity index**	1.8±0.4	1.7±0.5	1.6±0.4	2.6±0.3	1.4±0.4	1.5±0.5	
	**No. of Medications**	6.3±2	6±3	6.0±3	5.9±4	3.6±2	4.1±2	*

§ Data represented as mean ± standard deviation, unless otherwise stated.

* p<0.05 for differences between settings; differences between gender didn't reach any statistical significance.

Anorexic subjects were older than their nonanorexic counterparts (83±7 versus 76.6±8 years; p<0.05), showing a higher level of comorbidity (p<0.05). Gastrointestinal symptoms were more frequent in anorexic subjects although not reaching a statistically significant level. Anorexic older persons were shown to be more dependent on other people in the activities of daily living (p<0.05), to purchase food (84 versus 72.4%; p<0.05) and to cook food (81.1 versus 67.2%; p<0.05). Moreover, 15.7% of anorexic subjects (versus 0% in normal eating subjects; p<0.05) complained a different perception of the taste of foods from the usual taste; these subjects more frequently had an impairment of the masticatory function (reduced number of teeth: 7.1±9 versus 12±11; p<0.05) and swallowing was more severely impaired in anorexic subjects (11.9 versus 3.8%; p<0.05). Geriatric Depression Scale and Cornell Scale scores, the deterioration of cognitive status shown by MMSE (18.5±9 versus 23.8±5), and the level of comorbidity were higher (p<0.05 in all cases) (table 2).

**Table 2:** Baseline Characteristics of the Sample According to the Diagnosis of Anorexia§.

				A	NES	
**Subjects**	Males			41 (19.7%)	167	
	Females			71 (22.3%)	248	
**Age**	Years			83±7*	76.6±8	
**Clinical status**	Comorbidity index			2.4±2*	2.1±2	
	Severity index			1.6±0.5	1.9±0.6	
**Gastrointestinal symptoms**	Constipation (%)			21.7	21	
	Diarrhea (%)			27.9	20,7	
	Epigastric pain (%)			24,2	20,4	
**Cognitive status**	MMSE (score)			18.5±9*	23.8±5	
**Depression**	GDS (score)			6.3±5*	4.3±4	
	Cornell depression scale (score)			12.1±7*	8.7±7	
**Functional status**	IADL (score)			4.2±5*	7.9±6	
	ADL (>2 lost functions) (%)			55.5*	31.8	
**Need for assistance**	Shopping (%)			84*	72.4	
	Cooking (%)			81.1*	67.2	
**Masticatory function**	Residual teeth			7.1±9*	12±11	
**Swallowing function**	post test SpO2			94.8±3*	96±2	
	Difficulty in swallowing (%)			11.9*	3.8	
**Taste**	Different perception (%)			15.7*	0	
**Nutritional status**	MNA (score)			13±5*	21.9±5	
	Anthropometry	BMI (kg/m^2^)		23±4*	26.7±4	
		TSF (mm)	M	7.1±2*	9.6±3	
			F	12.8±5	15.4±6	
		AC (cm)	M	23±3*	28±3	
			F	24±4*	27.5±4	
		AMC (cm)	M	21±2*	25±2	
			F	21±3*	22.6±3	
**Laboratory parameters**	Prealbumin (mg/dl)			18.4±9	21.5±14	
	Albumin (g/dl)			3.4±0.6	3.5±0.5	
	Transferrin (mg/dl)			196.5±56	204.4±59	
	CRP (mg/l)			24.2±45*	12.9±16	
	Total cholesterol (mg/dl)			170.2±44*	208.7±36	
	Ghrelin (pg/ml)			243.2±178	234.6±217	
	Leptin (ng/ml)			8±19	13±19	
	IL-6 (ng/ml)			3.8±5	5.2±13	

§ Data represented as mean ± standard deviation, unless otherwise stated; * p<0.05.

Abbreviations: A: anorexia; NES: normally eating subjects; MMSE: Mini Mental State Examination; GDS: Geriatric Depression Scale; ADL: Activities of Daily Living; IADL: Instrumental Activities of Daily Living; BMI: Body Mass Index; MNA: Mini Nutritional Assessment; TSF: triceps skinfold; AC: arm circumference; AMC: arm muscle circumference; CRP: C-reactive protein.

The multivariate regression analysis was performed using only the independent variables significantly correlated with anorexia (outcome variable) in the univariate analysis: age, comorbidity index, MMSE and GDS score, ADL and IADL, number of residual teeth, different perception of taste, SpO_2_. In the block model of the regression analysis all the selected variables were included and R^2^ and R^2^adj of the model were respectively 0.53 and 0.3. The strength of association between anorexia and independent variables reached a statistical significant level (p<0.05) only for IADL (r = 0.4) and for the number of residual teeth (r = 0.35).

In anorexic subjects a more compromised nutritional status was observed, as shown by the MNA score (13±5 versus 21.9±5; p<0.05) and by lower anthropometric parameters (TSF, AC, AMC, BMI; p<0.05) than non-anorexic subjects. Biochemical parameters also confirmed the presence of malnutrition (even if differences were not statistically significant between anorexic and nonanorexic subjects); especially, C- reactive protein (CRP) concentrations were higher (24.2±45 versus 12.9±16 mg/l; p<0.05), and total serum cholesterol levels (170.2±44 versus 208.7±36 mg/dl; p<0.05) were significantly different in anorexic individuals. No difference was found for ghrelin, leptin, and IL-6 related to anorexia (table 2).

The frequency of consumption for all the food groups was generally reduced in anorexic subjects, even if it was not homogeneous considering different food groups. In particular, a reduction in the consumption of the groups “meat, fish, eggs” and “fruit and vegetables” was more frequently observed in anorexic elders when compared to the respective recommended daily intake; a more slight difference was described for cereals, whereas no difference was found in milk consumption comparing anorexic subjects to their nonanorexic counterparts. Consumption of oral supplements and reduced consistency food (e.g. pureed foods), was more frequent in anorexic subjects (41.3 versus 5.9% and 40.9 versus 21.7%, respectively; p<0.05). An association between the nutritional status and the frequency of consumption of certain food groups was pointed out: nutritional parameters were significantly better in subjects who ate a sufficient amount of “fruit and vegetables” and “meat, fish and eggs”. In particular, MNA scores were significantly better in subjects who had a regular intake of these food groups: 17.4±6 versus 12.1±4 (p<0.05) for “fruit and vegetables” and 18±6 versus 12.6±5 (p<0.05) for “meat, fish and eggs”. Besides, an adequate intake of milk was not associated to better nutritional parameters: 16.2±7 versus 14.2±6, respectively for regular and reduced/absent consumption (p>0.05).

Diet variety (defined as the number of food groups introduced daily according to the recommended amounts) was also taken into account (table 3), and it emerged that a more varied diet was related to a better nutritional status, especially in terms of BMI and MNA score (p<0.05). The intake of oral supplements and reduced consistency food was more frequent as the diet was less varied (p<0.05).

**Table 3:** Diet Variety and Nutritional Status in Anorexic Subjects§.

Number of food groups° regularly consumed		1	2	3	4	p
**Age (years)**		83.4±6	80.2±8	78.4±8	76.2±8	0.000
**Clinical status**	Comorbidity index	3.2±2	2.4±2	2.4±2	2±1	0.009
	Severity index	1.6±0.4	1.6±0.6	1.6±0.5	2.1±0.5	NS
**Gastrointestinal symptoms**	Constipation (%)	38.5	31.4	37.4	37.1	NS
	Diarrhea (%)	15.4	8.6	6.5	8.1	NS
	Epigastric pain (%)	46.2	32.9	27.3	18.8	0.01
**Cognitive status**	MMSE (score)	16.3±6	18.8±9	23±6	23±6	0.000
**Depression**	GDS (score)	1.2±0.4	1.4±0.5	1.3±0.4	1.3±0.4	NS
	Cornell depr. scale (score)	1.2±0.4	1.3±0.4	1.2±0.4	1.2±0.4	NS
**Functional status**	IADL (score)	4.5±5	5.2±5	8±5	6.3±5	0.002
	ADL (>2 lost functions) (%)	69.2	50	29,5	39	0.002
**Need for assistance**	Shopping (%)	81.8	78.4	73.1	85.5	NS
	Cooking (%)	72.7	74.5	68	84.8	NS
**Masticatory function**	Residual teeth	9.3±11	8.5±10	11.5±11	11.1±11	Ns
**Swallowing function**	post test SpO2	96±2	95±4	95.2±2	96.2±1	NS
	Difficulty in swallowing (%)	0	21.1	8.1	0	NS
**Taste**	Different perception (%)	16.7	11.1	18.5	0	NS
**BMI (kg/m^2^)**		22.8±2	23.6±3	24.1±4	27±6	0.038
**MNA (score)**		11.8±6	14.1±5	16.4±7	19.4±7	0.005
**Prealbumin (mg/dl)**		11.6±3	19,3±6	18.8±12	21.3±12	NS
**Albumin (g/dl)**		3.4±0.4	3.4±0.6	3.4±0.6	3.5±0.4	NS
**Transferrin (mg/dl)**		184±34	216±68	184±55	204±53	NS
**CRP (mg/l)**		15.9±19	20.5±36	21.2±38	22.4±50	NS
**Cholesterol (mg/dl)**		182±41	183±41	163±46	189±45	NS
**Oral Supplements (%)**		38.5	25.4	14.9	6.6	0.000
**Reduced consistency foods (%)**		35.4	34.7	20.4	11.5	0.001

° Food groups considered: “milk and dairy products”, “meat, fish, and eggs”, “cereals and derivatives” “fruit and vegetables”. The dimension of portions for each food were based on Italian Recommended Daily Allowances (LARN: Livelli di Assunzione Raccomandati di Energia e Nutrienti) (23).

§ Data represented as Mean ± Standard deviation, unless otherwise stated.

Abbreviations: MMSE: Mini Mental State Examination; GDS: Geriatric Depression Scale; ADL: Activities of Daily Living; IADL: Instrumental Activities of Daily Living; BMI: Body Mass Index; MNA: Mini Nutritional Assessment; CRP: C-reactive protein.

Moreover, diet variety was also related to age (it was higher in younger subjects; p<0.05), clinical status (a less varied diet was found in subjects with a higher level of comorbidity or with epigastric pain; p<0.05), autonomy level and cognitive status (functional and cognitive impairment was linked to a worse index of diet variety; p<0.05). On the other hand, no correlation was found among the diet variety and mood, number of medications, constipation, diarrhea, impaired taste sensitivity, swallowing and masticatory function, ghrelin, leptin and interleukin-6 levels. A multivariate regression analysis was performed using only the independent variables significantly correlated with diet variety (outcome variable) in the univariate analysis: age, comorbidity index, MMSE score, ADL and IADL. In the block model of the regression analysis all the selected variables were included and R^2^ and R^2^adj of the model were respectively 0.11 and 0.09. The strength of association between diet variety and independent variables reached a statistical significant level (p<0.05) only for age (r = 0.24) and MMSE (r = 0.26).

Differences between participants from each setting were not found considering diet variety and the association of it to explanatory variables (data not shown).

## Discussion

In our study results showed that anorexia in the elderly does not mean only a global reduced food intake, but it is characterized by an eating pattern in which some food groups (“meat, eggs and fish” and “fruit and vegetables”) are more penalized than other food categories (milk and cereals).

A number of previous studies highlighted that in older persons a progressive reduction of the daily calorie intake occurs, mainly due to the decrease in consumption of high fat foods [35]. In our study population the reduced daily calorie intake was ascribed to a decrease in the consumption of food groups like “fruit and vegetables” as well as “meat, eggs and fish”, the latter representing the principal source of high quality proteins.

Accumulating evidence indicates the importance of protein intake in the health status of older adults, especially as it relates to muscle and bone, which are well known to decline with age. In particular, observational studies in elders, including the Health ABC study, showed that a higher protein intake was associated with higher muscle mass and a reduced loss of lean mass over time [36], [37]. Moreover, nitrogen balance studies in the elderly reported conflicting results concerning the current recommendation for protein intake of healthy elderly subjects; some studies suggested that not all elderly can achieve a nitrogen balance with a protein intake of 0.8 g/kg body weight/day, particularly if energy supply is not adequate [38].

Noteworthy, in our study fruit and vegetable consumption was lower than recommended amounts (less than 7 days/week) in at least one third of subjects with anorexia. The consequence is a reduced intake of nutrients and bioactive substances whose positive effect was widely demonstrated in modulating some phenomena strictly linked to the aging process (such as *inflammaging*, oxidative stress, gut microbiota balance) [39]. A greater consumption of fruit and vegetables is associated with better food habits [40], and the literature suggests that these foods may be beneficial to elderly subjects, as well as a number of cross- sectional studies found a positive correlation between fruit and vegetables intake and bone mineral density [41], and a negative correlation with the onset of neurodegenerative disorders [42].

Fruit and vegetables are important sources of micronutrients, including vitamins E and C, that play crucial roles in optimal cell functioning (as components of biological membranes, as co- substrates for several enzymes, involved in the antioxidant defense of the cell, contributing to the redox status of the cell). Consistently with our study, previous studies found that elderly subjects frequently did not meet the official recommendations for fruit and vegetable intake. Therefore, especially the intake of fiber, vitamins (vitamins E, D, B1, B12 and folic acid) and micronutrients (iron, potassium and calcium) and bioactive compounds (polyphenols, carotenoids, phytosterols) was frequently below the recommended dietary allowances [43], . Although an optimal diet for frail elderly subjects (with increased comorbidity and reduced autonomy levels as in our sample) has not yet been established, it is likely that an adequate intake of fruit, vegetables and related micronutrients may be beneficial also in this population.

The present study highlighted a worse nutritional status associated to a decreased ingestion of all the food groups; with respect to milk and dairy products, we observed a relatively high frequency of consumption even in subjects with malnutrition.

Milk and dairy products are rich in calcium and previous studies suggested that there may be an association between functional disability and low calcium intake because of its role in bone health, bone structure and function, peak bone mass, osteoporosis, and osteoporosis- related fractures [45], [46]. Moreover, milk and dairy products consumption in older men was associated with a significantly reduced risk of disability [47]. Although it is biologically plausible that milk and dairy product consumption can reduce the risk of functional disability, the results of our study are partially inconsistent with the literature. We can argue that the adequate frequency of consumption of milk and dairy products in our study (one serving of “milk and dairy products” once a day, based on the Italian Recommended Daily Allowances [23] was subsequent to a reduced consumption of other food groups. Paradoxically, milk seems to be a marker of a reduced global dietary intake in both qualitative and quantitative terms.

A varied diet is a diet that encompasses all the food groups. According to guidelines for a healthy and correct nutrition, a varied diet allows to achieve the recommended amounts of macronutrients, providing energy, and essential micronutrients. Hence, a high nutritional quality of diet is a pivotal prerequisite for maintaining a good nutritional status. In the elderly population, exclusion of one or more food groups may lead to a less varied diet, worsening the nutritional status [48].

Consistently with existing literature indicating a preference for a monotone diet in elders, the evaluation of the variety of diet in our study showed a trend in the whole sample of subjects in training on a poor quality diet, just based on few repetitive foods. In addition, our results confirm the ascertained association between a low variety diet and poor nutritional status (assessed by nutrient intake, biochemical measures, and body composition measures). As stated in other studies, this may represent a higher morbidity risk factor [48].

We verified the relationship between the variety of the diet and clinical and functional variables affecting nutritional intake. Following the classification proposed by Nieuwenhuizen et al., these factors can be divided in three categories: personal factors (social, physiological and psychological changes); food factors affecting gastric distension and emptying; environmental factors (eating with a family member, encouragement by caregivers, and a pleasant eating environment) [49]. Our results reported a higher diet variety in younger subjects, whereas a low variety diet was observed in subjects having a higher comorbidity index, a lower autonomy level and an impaired cognitive status. Inconsistently with previous papers, no association was found out between diet variety and mood, number of medications, diarrhea, constipation, impaired taste perception, swallowing, and masticatory function. Of note, in our study population the poor variety of diet was well reflected by the use of reduced consistency foods (40.9%), oral supplements (41.3%) and milk (90%).

Otherwise, no significant differences in eating patterns were observed in respect of hormones and cytokines involved in feeding and hunger control. In the literature there is some evidence that ghrelin production and sensitivity is altered during aging, and it may be one of the potential explanations of the beginning of the phenomenon of anorexia of aging. Moreover, leptin and inflammation appear to be potential determinants of nutritional status [50], [51]. In our sample the lack of correlation between eating patterns and cytokines and ghrelin may be due to the large variability in measurements of circulating leptin and ghrelin concentrations, perhaps reflecting the heterogeneity of older persons living in different settings.

The major limitation to our study was represented by the difficulty in assessing taste and chewing ability. We tried to assess taste function using an ascending- series staircase methodology (stimuli presented were sucrose, sodium chloride, citric acid and quinine hydrochloride) and mastication function through a gum chewing test. Cognitive and functional impairment, on one hand, and depression and comorbidity on the other hand, prevented most subjects to complete the tasks, thus the obtained data were unreliable. Moreover we didn't consider water and fluids intakes. Although being beyond the aim of our study, these data can be considered very important in the evaluation and management of senile anorexia.

Considering the high prevalence of anorexia in the geriatric population and the great impact on nutritional status, further research should be prompted in order to establish an intervention protocol allowing the early diagnosis of anorexia of aging, aimed at identifying its causes and at optimizing treatment of anorexic elderly patients. Although the study was based only on geriatric subjects, it did not specifically address the issue of the effect of age on dietary patterns. Overall the results seem to suggest that clinically complex patients have a more limited dietary variety than their healthy peers. We can imagine to modulate nutritional treatments on the basis of the causes of anorexia: increased density meals for subjects with impaired peripheral satiety system, enriched flavoured or enhanced foods for subjects with a decline in taste or smell functions, immunomodulating agents (e.g. polyunsaturated fatty acids) in patients showing an increased cytokine production.
